# Burial Depth Effect of Crack on the *L*_cr_ Wave Acoustoelastic Coefficient for Stress Measurement of Laser Cladding Coating

**DOI:** 10.3390/ma13122823

**Published:** 2020-06-23

**Authors:** Bin Liu, Zhihao Zeng, Jiayang Gu, Shujin Chen, Peng He, Jinxiang Fang

**Affiliations:** 1 Material Science and Engineering, Jiangsu University of Science and Technology, Zhenjiang 212003, China; Binliudely@163.com (B.L.); iszengzhihao@163.com (Z.Z.); chenshujin7120@126.com (S.C.); 2 Institute of Marine Equipment, Jiangsu University of Science and Technology, Zhenjiang 212003, China; 3 State Key Laboratory of Advanced Welding and Joining, Harbin Institute of Technology, Harbin 150001, China; Binliu1983@163.com; 4 School of Mechanical Engineering, Guizhou University, Guiyang 550000, China; Fangjx6@gmail.com

**Keywords:** burial depth of crack, *L*_cr_ wave, laser cladding coating, stress, non-destructive evaluation

## Abstract

In this paper, the influence of burial depth of crack on stress measurement of laser cladding coating with the critical refracted longitudinal wave (*L*_cr_ wave) was discussed based on the *L*_cr_ wave acoustoelastic effect. The regular rectangular slots with different depths that were used to simulate the burial crack in coating was based on the equivalent theory. The experimental system including an ultrasonic wave generator, digital oscilloscope (2.5 GHz sampling rate), and two *L*_cr_ wave transducers (2.5 MHz center frequency) was used to collect the *L*_cr_ wave under different tensile loads, and the *L*_cr_ wave was denoised by using wavelet analysis technology, then the fracture morphology was observed using SEM. The results show that after the denoising by wavelet analysis technology, the signal-to-noise ratio of *L*_cr_ wave becomes bigger and the mean square deviation of *L*_cr_ wave becomes smaller. When the tensile load is within the turning point load, the difference in time of flight between *L*_cr_ wave increases linearly as the tensile load increases, and the deviation of the experimental results becomes obvious as the tensile load increases. When the tensile load is the same, as the burial depth of the slot increases, the nominal *L*_cr_ wave acoustoelastic coefficient decreases and tends to be stable gradually. At last, the experimental results are discussed based on the *L*_cr_ wave acoustoelastic effect and deformation theory, and it is analyzed that the uneven deformation caused by the interface in coating, anisotropic microstructure, and the burial crack is considered as the main reason.

## 1. Introduction

Stress plays an important role in the performances of coatings, so finding an effective method for stress measurement of coating appears to be particularly urgent. Generally speaking, the stress measurement method of coating can be divided into two categories, including the destructive method (such as drilling-hole method, tensile method, etc.) and non-destructive method (such as optical method, ultrasonic wave method, X-ray diffraction method, magnetic method, etc.) [1,2,3,4,5,6,7]. Due to non-destruction, convenient operation, safety, etc., the scholars’ attention is attracted by the non-destructive technology, and its applications on stress evaluation also have been reported. While it should be noted that there is no absolute method for satisfying all the requirements of stress measurement, because there are lots of factors, including the characteristics, geometric shape, surface condition, etc., that should be taken into account to choose a method [8,9,10,11]. Compared with other non-destructive methods, the ultrasonic wave method has some significant advantages for evaluating stress, such as low cost, online measurement, operational safety, etc., so it is widely used in some industrial fields, including a pipeline, pressure vessel, etc.

The critical refracted longitudinal wave (*L*_cr_ wave) is one kind of ultrasonic wave, and its propagation velocity is more sensitive to stress than other kinds of ultrasonic waves, so the *L*_cr_ wave is usually employed to measure the stress of the plate. The *L*_cr_ wave acoustoelastic effect, which is the theoretical basis for stress measurement with *L*_cr_ wave, is a weak effect, it means that as stress changes, the change on the propagation velocity of *L*_cr_ wave is not very obvious, so the *L*_cr_ wave acoustoelastic formula is deducted by Husson and Ditri, and the propagation velocity of the *L*_cr_ wave is replaced by the difference in time of flight between the *L*_cr_ wave [12,13]. Based on that, the experimental studies and theoretical studies were designed and carried out. For example, the stress evaluation of different materials [14,15,16,17,18,19], including the welded joint, carbon steel, aluminium alloy, etc., and its influence factors [20,21,22,23,24,25], including the temperature, coupling state, microstructure etc., were discussed. It could be known that although lots of researches on different materials and influence factors had been discussed, the study on coating stress measurement was rare. Therefore, authors discussed the stress measurement of coating with the *L*_cr_ wave, and some findings were received, but there was no report about the influence of coating defect, which was very important and cannot be ignored, on stress measurement. It was analyzed that the noise caused by the coating microstructure and the simulation method for coating defect were the main reasons. Thus, finding a method for evaluating the influence of coating defect on stress is important and needs to be solved urgently.

Burial crack is a type of defect in coating, and its burial depth is very important for the coating stress. Thus, the stress of laser cladding coating with burial crack was measured with the *L*_cr_ wave method, and the influence of crack burial depth on stress was discussed, then the relation of the *L*_cr_ wave acoustoelastic coefficient and burial depth of crack was attempted to be determined in this paper. The detailed experimental procedures were described as follows: The regular rectangular slots with different burial depths, which were used to simulate the burial crack in coating, were machined in one surface of the sample, and then the calibration test of the *L*_cr_ wave acoustoelastic coefficient was carried out. Using the cross-correlation theory and denoising theory, the *L*_cr_ wave of laser cladding coating was analyzed, so the relation of the *L*_cr_ wave acoustoelastic coefficient and burial depth of crack was received. At last, the experimental results were discussed based on the *L*_cr_ wave acoustoelastic theory and elastic-plastic deformation theory of material.

## 2. Experimental Material and Methods

### 2.1. Experimental Material

**Figure 1:**
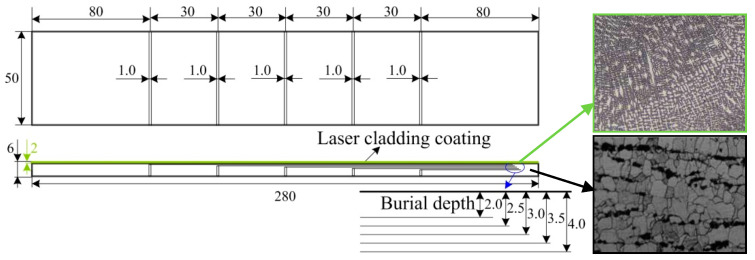
Sketch map of laser cladding Fe314 alloy coating with burial cracks.

In this study, the base metal is Q235 steel, and the cladding material is Fe314 alloy power. The main process parameters for preparing the laser cladding Fe314 alloy coating are as follows: The laser power is 1.7 kW, the cladding velocity is 2 mm/s, and the feeding rate of powder is 16 g/min, respectively. To simulate the burial crack in coating, the regular rectangular slots with different depths were machined in the surface of Q235 steel, as shown in Figure 1. The widths of all the slots are 1.0 mm, and the burial depth of slots are 2.0, 2.5, 3.0, 3.5, and 4.0 mm, respectively. After that, all the coating samples are heat treated for relieving initial stress using the WZS-20 vacuum furnace (AOKE, Hangzhou, China), and its vacuum heat treatment parameters are listed in Table 1.

### 2.2. L_cr_ Wave Acoustoelastic Theory

**Figure 2:**
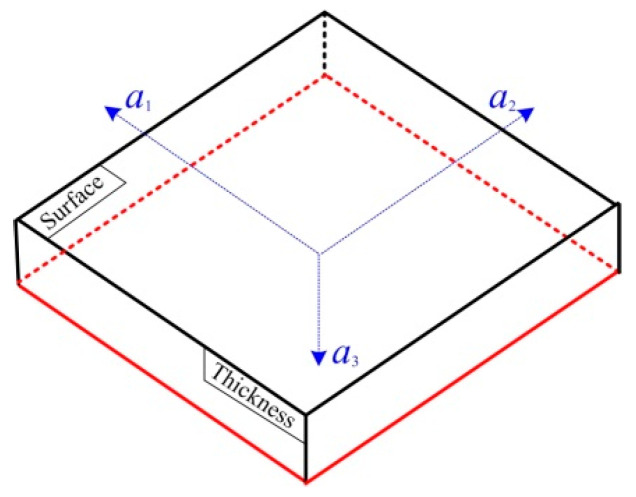
The three-dimensional plane in the orthogonal rectangular coordinate axis.

Under the definition of the three-dimensional plane of a medium in the orthogonal rectangular coordinate axis, shown in Figure 2, the complete tensorial description of the *L*_cr_ wave acoustoelastic effect was deduced and expressed as follows [26,27]:

(1) $$\frac{V_{L}(\theta )-V_{L}^{0}}{V_{L}^{0}}=(\frac{K_{1}+K_{2}}{2})(\sigma_{11}+\sigma_{22})+(\frac{K_{1}-K_{2}}{2})(\sigma_{11}-\sigma_{22})\cos(2\theta )$$

where *θ* was the angle between the propagation direction of *L*_cr_ wave and *a*_1_ direction, $V_{L}^{0}$ was the propagation velocity of *L*_cr_ wave in an unstressed medium, *K*_1_ and *K*_2_ were *L*_cr_ wave acoustoelastic coefficients along the *a*_1_ and *a*_2_ direction, *σ*_11_ and *σ*_22_ were the principal stress along the *a*_1_ and *a*_2_ direction.

If the propagation direction of *L*_cr_ wave was along the *a*_1_ direction, which was the loading direction, Equation (1) could be simplified as:

(2) $$\frac{V_{1}-V_{L}^{0}}{V_{L}^{0}}=K_{1}\sigma_{11}+K_{2}\sigma_{22}$$

where *V*_1_ was the propagation velocity of *L*_cr_ wave along the *a*_1_ direction.

Based on the *L*_cr_ wave acoustoelastic theory, the result of *K*_1_>>*K*_2_ could be accepted [28], thus Equation (2) could be simplified as:

(3) $$\frac{V_{1}-V_{L}^{0}}{V_{L}^{0}}=K_{1}\sigma_{11}$$

When the propagation distance of *L*_cr_ wave was fixed, Equation (3) could be simplified as:

(4) $$\Delta t=K_{1}\sigma_{11}=K\cdot \sigma$$

where Δ*t* was the difference in time of flight between *L*_cr_ wave, and *σ* was the stress.

Since the burial depths of cracks were different, the stress *σ* was replaced by the tensile load. Thus, Equation (4) could be written as follows:

(5) $$\Delta t=K\cdot \sigma =K\cdot \frac{F}{S}=K^{\prime }\cdot F$$

where *K′* was the nominal *L*_cr_ wave acoustoelastic coefficient, *F* was the tensile load, and *S* was the cross-section area of the coating sample. 

From Equation (5), it can be known that when Δ*t* and *F* are determined, the *K′* can be received, then the relation of the burial depth of crack and *K′* can be determined.

### 2.3. The Cross-Correlation Theory

The cross-correlation function was usually used to calculate the difference in time of flight, and it can be expressed as:

(6) $$R_{xy}(\tau )=\frac{1}{T}\int_{0}^{T}x(t)y(t+\tau )dt$$

where *R_*xy*_*(**τ**) was the cross-correlation function, *τ* was the difference in time of flight between *x*(t) and *y*(t), and *T* was the signal period.

To eliminate the influence of the *L*_cr_ wave amplitude on the difference in time of flight between signals, the cross correlation function was normalized and discretized [29], and it was defined as the cross correlation coefficient function *ρ*_*xy*_(**τ**) and written as follows:

(7) $$\rho_{xy}(\tau )=\frac{\sum x(i)\cdot y(i)-\sum x(i)\cdot \sum y(i)/n}{\sqrt{\left[\sum x^{2}(i)-(\sum x(i){)}^{2}/n\right]}\cdot \sqrt{\left[\sum y^{2}(i)-(\sum y(i){)}^{2}/n\right]}}\;\;\;(i=1,\;2,\;3\;\ldots \;n)$$

where *ρ*_*xy*_(**τ**) was the cross-correlation coefficient function, *x*(*i*) and *y*(*i*) were two different signals, and *n* was defined as the step length in this paper. It could be known that when the maximum of *ρ*_*xy*_(*τ*) was received, the difference in time of flight between signals could also be determined.

### 2.4. Experimental System for Stress Evaluation

The experimental system for stress measurement of laser cladding Fe314 alloy coating with the *L*_cr_ wave consists of an ultrasonic wave generator (Olympus 5072 PR, OLYMPUS, Tokyo, Japan), digital oscilloscope (Tektronix DOP3034B, TEKTRONIX, Beaverton, OR, USA), and *L*_cr_ wave transducers, including one transmitting transducer and one receiving transducer, which are designed by the authors. To meet the requirement of *L*_cr_ wave sampling, the sampling rate is 2.5 GHz. For the best distance between two *L*_cr_ wave transducers, the energy attenuation experiment of *L*_cr_ wave propagating in coating was tested, and the results showed that when the center frequency of *L*_cr_ wave is 2.5 MHz, the best propagation distance of *L*_cr_ was 30 mm. In addition, the pressure holder of the *L*_cr_ wave transducer was designed and employed to keep a constant pressure between *L*_cr_ wave transducers and the coating surface. The calibration test of the *L*_cr_ wave acoustoelastic coefficient was carried out using the SANS-CMT5205 static tensile testing machine (MTS-China, Shanghai, China).

## 3. Results and Discussion

### 3.1. Relation of the L_cr_ Wave Acoustoelastic Coefficient and Burial Depth of Crack

**Figure 3:**
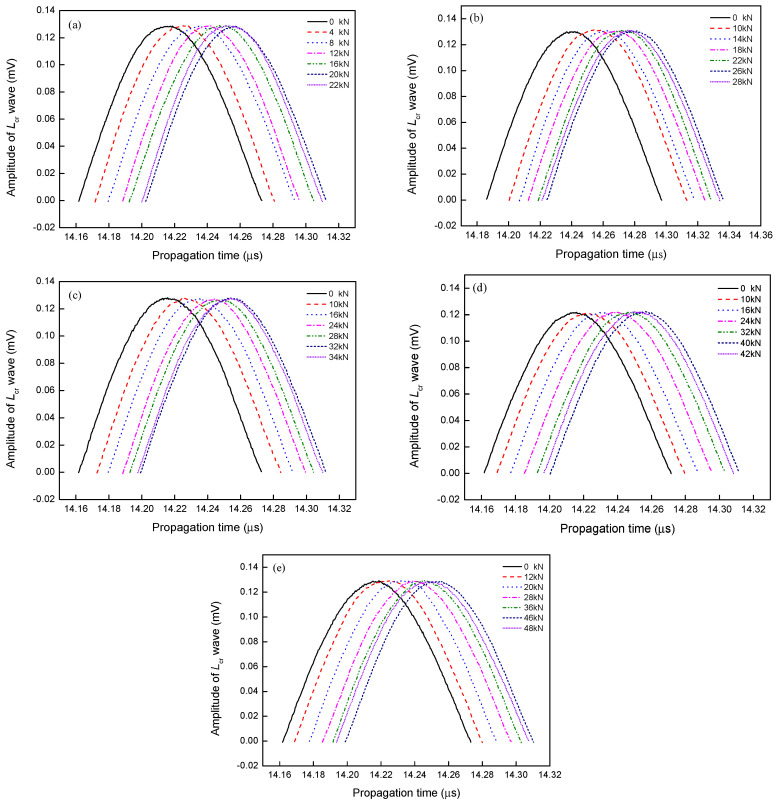
*L*_cr_ wave of coating with different burial rectangular slots (**a**) 2.0, (**b**) 2.5, (**c**) 3.0, (**d**) 3.5, (**e**) 4.0 mm.

To determine the *L*_cr_ wave acoustoelastic coefficient, the *L*_cr_ wave of coating with a rectangular slot was collected with the uniaxial static tension experiment. During the test, the maximal tensile load was the yield load of coating, and the loading rate was 0.5 kN/s. To collect the *L*_cr_ wave of coating under different tensile loads, the preloads were determined beforehand, and each preload was held about 60 s, then the *L*_cr_ wave was collected. In that process, the *L*_cr_ wave transducers were placed on the top of the rectangular slot, and the *L*_cr_ wave propagated along the loading direction. To avoid the influence of couplant between the *L*_cr_ wave transducer and coating surface, the pressure on the *L*_cr_ wave transducer was kept stably, and the *L*_cr_ wave for each tensile load was collected five times repeatedly. Contrasting the *L*_cr_ wave, it could be seen that under the same tensile load, there was almost no difference in the propagating time of *L*_cr_ wave, it meant that the influence of the couplant on *L*_cr_ wave could be ignored. In this paper, the first *L*_cr_ wave of coating was analyzed and shown in Figure 3.

From Figure 3, it can be seen that for different burial depths, the change of *L*_cr_ wave along the time axis is very similar. As the tensile load increases, the *L*_cr_ wave gradually moves to the right along the time axis, it means that the propagation time of *L*_cr_ wave becomes longer. Since the propagation distance of *L*_cr_ wave is the same, the propagation velocity becomes lower as the tensile load increases. However, it should be noted that when the tensile load reaches a certain value, which is defined as a turning point load in this paper, as the tensile load increases, the change of *L*_cr_ wave is not continually regular as before, so the change on the propagation velocity of *L*_cr_ wave is irregular as the tensile load increases further. In addition, when the tensile load changes to the same, the change on the propagation time of *L*_cr_ wave is different as the burial depth changes gradually, while it is not very obvious. For that reason, the calculation accuracy of the difference in time of flight between *L*_cr_ wave seems very important. However, from the *L*_cr_ waveform, it can be seen that the noise is obvious, so the difference in time of flight is affected, which has been proven by the previous study. Therefore, the *L*_cr_ wave is denoised firstly, and the denoising parameters are optimized.

**Figure 4:**
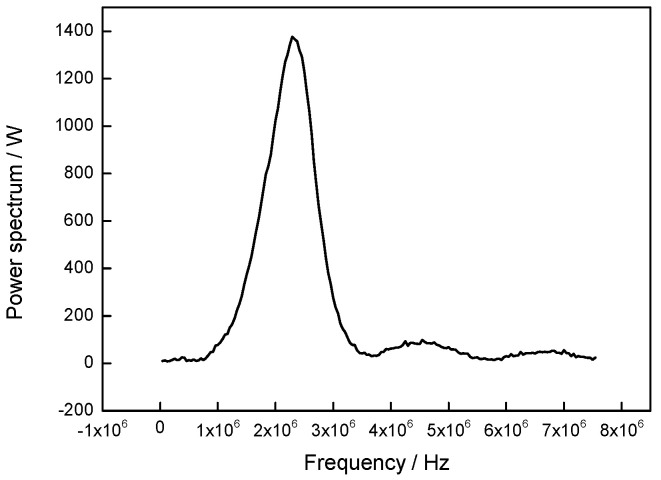
The frequency domain of *L*_cr_ wave.

The wavelet analysis method is a commonly used method for denoising, and the main parameters of denoising include the mother wavelet, decomposition level, and the threshold method, which are discussed in this paper. From the waveform similarity, the db6 wavelet was chosen as the mother wavelet. To get the distribution of frequency of *L*_cr_ wave, the frequency domain of *L*_cr_ wave of coating was extracted by using the FFT method, and the results were shown in Figure 4. 

**Figure 5:**
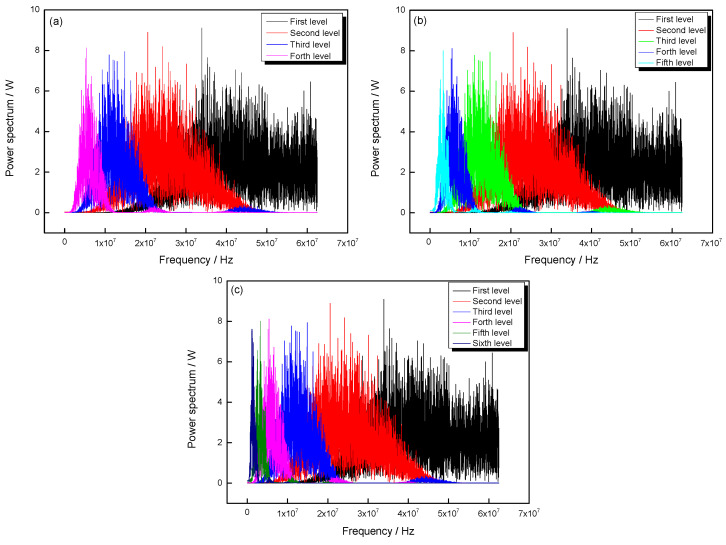
The frequency domain of *L*_cr_ wave with different decomposition levels. (**a**) Four levels, (**b**) five levels, (**c**) six levels.

From Figure 4, it can be seen that under the 3 db theory, the main frequency domain of *L*_cr_ wave is in the range of 1.98~2.63 MHz. Compared with the center frequency of *L*_cr_ wave, the frequency domain distribution of noise can be determined. Based on that, the influence of decomposition level on the denoising result was discussed, and the results were shown in Figure 5.

**Figure 6:**
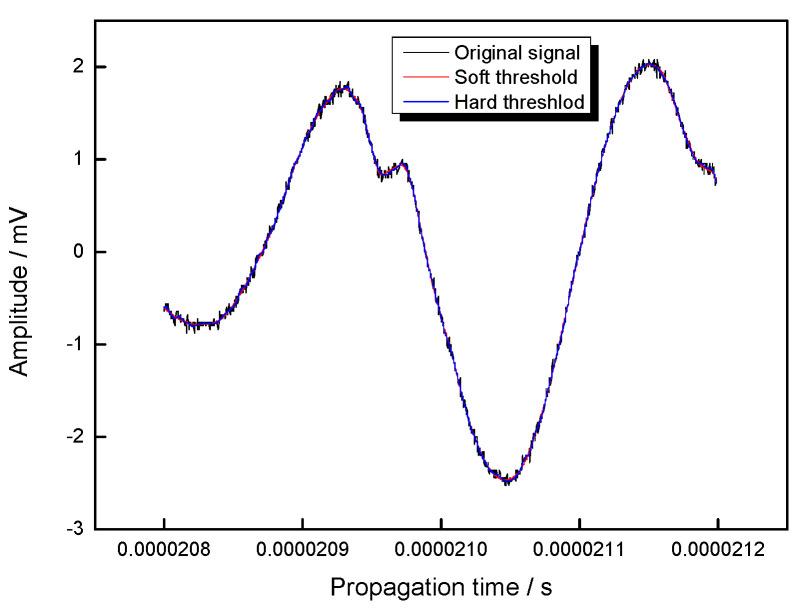
*L*_cr_ wave after denoising.

Figure 5 shows that when the decomposition level is four, the noise cannot be separated from *L*_cr_ wave. When the decomposition level is five, the noise can be mainly separated from *L*_cr_ wave. When the decomposition level is six, the frequency domain of *L*_cr_ wave almost does not change compared with that of five levels. Thus, the five levels can meet the denoising requirement of *L*_cr_ wave. Based on that, the threshold method was discussed, and the result was shown in Figure 6.

From Figure 6, it can be seen that compared with the denoising signal by using a soft threshold, the denoising waveform by using a hard threshold is smoother. In order to quantitatively evaluate the denoising result, the signal-to-noise ratio and the mean square deviation of *L*_cr_ wave after denoising were calculated and compared, and it was shown in Table 2.

**Figure 7:**
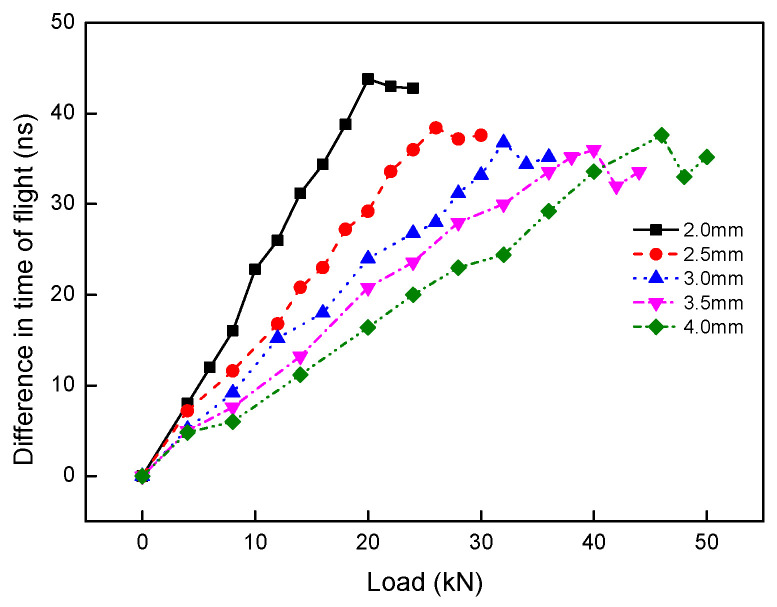
The relation of difference in time of flight and tensile load.

From Table 2, it can be known that compared with the results of the soft threshold, the signal-to-noise ratio of the hard threshold is higher, and its mean square deviation is lower. The denoising theory indicates that a higher signal-to-noise ratio and lower mean square deviation means the better the denoising effect, so the hard threshold method is adopted in this paper. After that, the difference in time of flight between *L*_cr_ wave was determined by Equation (7). The previous studies indicated that the step length affected the accuracy of the difference in time of flight between signals, and one cycle was the most optimal step length [30]. Based on that, the difference in time of flight between *L*_cr_ wave was determined, and its relation with the tensile load was shown in Figure 7.

From Figure 7, it can be seen that for different burial depths of slots, the whole trend of the difference in time of flight when the tensile load increases is very similar, but there are also some differences. For the similarities, the difference in time of flight increases linearly as the tensile load increases, which is consistent with the *L*_cr_ wave acoustoelastic theory. While when the tensile load reaches the turning point load, the difference in time of flight changes nonlinearly as the tensile load increases further. In addition, the deviation of curves from linearity becomes more obvious as the tensile load increases. For the differences, when the tensile load is the same, the difference in time of flight becomes lower gradually as the burial depth increases. For the above results, it is analyzed that the non-uniform distribution of stress caused by the slot and coating microstructure is the main reason.

**Figure 8:**
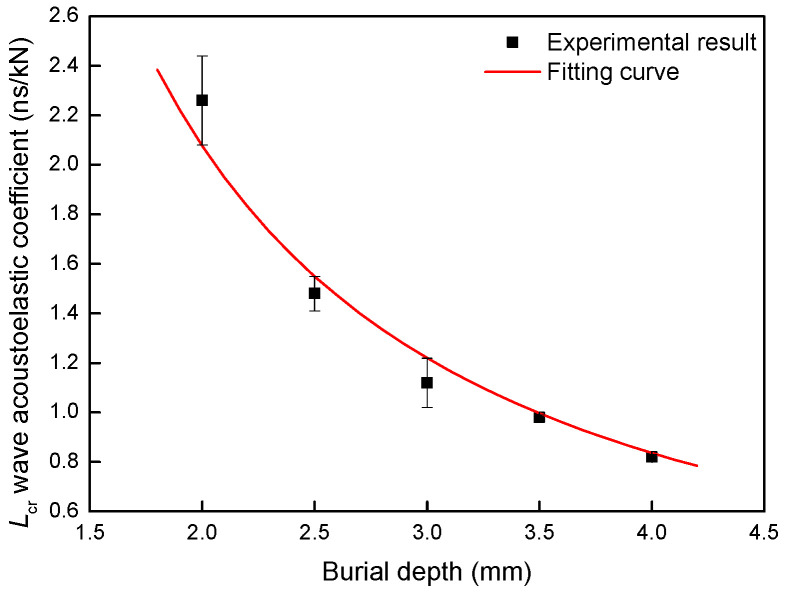
Curve of fitting coefficient and burial depth of slot.

Based on Equation (5), the difference in time of flight and tensile load within the turning point load was fitted with a linear function, so the relation of the fitting coefficient and burial depth of slot was received and shown in Figure 8.

As shown in Figure 8, it can be seen that as the burial depth of slot increases, the fitting coefficient decreases and tends to be stable gradually. To quantitatively describe the burial depth effect of slot on stress evaluation with *L*_cr_ wave, the result was fitted by the power function, and it could be expressed as:

(8) $$K^{\prime }=5.1562\cdot D^{-1.3118}$$

where *K*′ was the fitting coefficient, and *D* was the burial depth of slot. 

### 3.2. Discussion and Analysis

First, from the interaction of *L*_cr_ wave and the medium, it can be known that the interface in coating and the anisotropic microstructure of coating caused by directional solidification are the main reasons for noise in the *L*_cr_ wave. The preparation method of coating shows that there are some interfaces between layer and layer, and it appears to be a circular arc, so the reflection of *L*_cr_ wave on the interface is very irregular, and the noise in *L*_cr_ wave is obvious.

**Figure 9:**
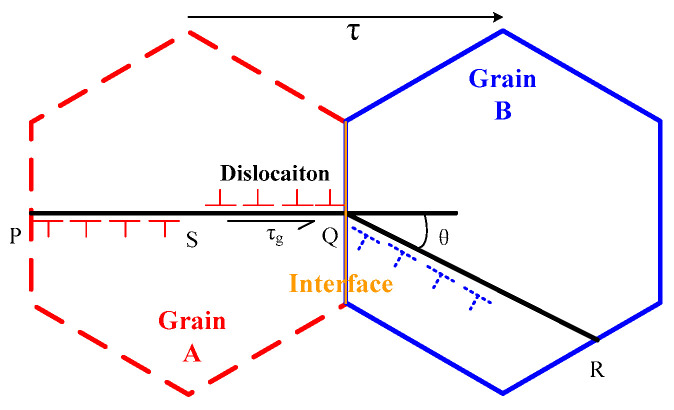
Dislocation accumulation model for plastic deformation.

Second, the *L*_cr_ wave acoustoelastic theory is the base for the experiment, so when the tensile load is within the turning point load, the difference in time of flight between the *L*_cr_ wave of coating varies almost linearly as the tensile load increases. However, it should be emphasized that the isotropic media is the premise of the *L*_cr_ wave acoustoelastic theory, while the microstructure of coating is obviously anisotropic, shown in Figure 1, so the experimental result is not very consistent with the *L*_cr_ wave acoustoelastic theory. It is analyzed that during the loading process of coating, the macroscopic deformation of coating is still in the elastic stage, but some small zones may be in the state of plastic deformation, which is not consistent with the deformation premise of the *L*_cr_ acoustoelastic theory. As the tensile load increases, the plastic deformation area becomes bigger gradually, so the deviation of the experimental result becomes more obvious. To prove it, the dislocation accumulation model for explaining plastic deformation was employed, and its model was shown in Figure 9.

Figure 9 shows the process of plastic deformation caused by dislocation movement, an internal stress is generated and it can be expressed as follows:

(9) $$\tau_{g}=\frac{2L{\left(\tau -\tau_{i}\right)}^{2}}{Gb}$$

where *τ*_g_ is the internal stress, *τ* is the shear stress on PQ surface, *τ*_i_ is the resistance of dislocation in grain, *L* is the length of dislocation group, *b* is the Brinell vector of dislocation, and *G* is the shear modulus of metal.

**Figure 10:**
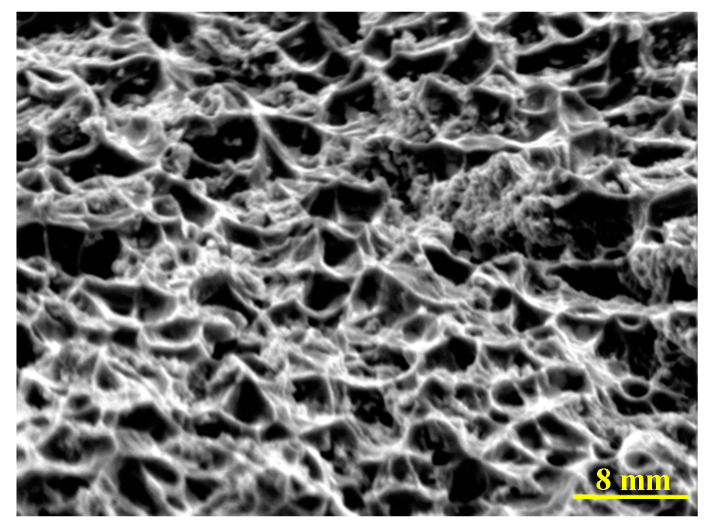
Fracture morphology of coating.

From Equation (9), it can be seen that as the dislocation number increases, the length of the dislocation group becomes longer, so the internal stress *τ*_g_ becomes bigger gradually. When the internal stress becomes higher than the stress for moving the dislocation group, the dislocation moves from grain A to grain B, it means the deformation of grain A is transferred to grain B, meanwhile the internal stress is released. During the deformation process, the release and concentration of stress repeats many times until the plastic deformation can be observed. Considering the coating microstructural characteristic, the dislocation may be pinned by the interface in coating and the anisotropic microstructure, it means the stress concentration is formed, and there is a higher stress. While as the tensile load increases, the pinning effect of interface and microstructure on dislocation can be broken, so the dislocations continue to move, it means the stress is released. Therefore, the internal stress state changes during the loading process of coating, so the relation of the difference in time of flight and tensile load is not strictly linear as Equation (5). For the above analysis, it should be noted that the dislocation movement with many repetition times requires a well deformation capacity, so the SEM image of the coating fracture was observed and shown in Figure 10.

From Figure 10, it can be seen that lots of dimples, which is the typical characteristic for a well plastic deformation capacity, appear in the surface fracture of coating, and means the above theoretical analysis is reasonable.

In addition, the stress concentration caused by the slot is another important reason for the experimental results. As well known, the stress concentration effect is a typical result caused by the crack, and the stress concentration effect becomes more obvious as the crack depth increases. It means that the stress concentration effect becomes more obvious as the burial depth of slot decreases in this paper, so when the tensile load is the same, the real stress becomes smaller as the burial depth of the slot increases gradually as shown in Figure 8.

## 4. Conclusions

In this paper, the influence of burial depth of crack on stress measurement of laser cladding coating with *L*_cr_ wave was discussed, and the experimental results were analyzed based on the *L*_cr_ wave acoustoelastic theory and deformation theory. The results could be concluded as that:

The structural interface and anisotropy of coating are the main reasons for the noise in *L*_cr_ wave. Using the wavelet analysis theory, the *L*_cr_ wave is denoised, and the optimal parameters are as follows: db 6, five decomposition levels, and hard threshold, respectively. For the coating deformation, it is not uniform because of the structural characteristics of coating, and it results in the difference between the experimental result and *L*_cr_ wave acoustoelastic theory, which is explained by the dislocation accumulation. In addition, the stress concentration effect caused by burial crack is another reason for the difference between the experimental result and theoretical result. For the results, it can be known that as the burial depth of crack increases, the *L*_cr_ wave acoustoelastic coefficient becomes smaller gradually.

## Figures and Tables

**Table 1:** Vacuum heat treatment technology.

Heating Rate/(°C·s^−1^)	Maximal Temperature/°C	Holding Time/Min	Cooling Mode
10	550	30	Furnace cooling

**Table 2:** The signal-to-noise ratio and the mean square deviation of *L*_cr_ wave after denoising.

Threshold Method	Signal-to-Noise Ratio	Mean Square Deviation
soft threshold	18.721	0.031927
hard threshold	19.202	0.030205
